# Synthesis of Antibacterial Gelatin/Sodium Alginate Sponges and Their Antibacterial Activity

**DOI:** 10.3390/polym12091926

**Published:** 2020-08-26

**Authors:** Yanyi Wen, Bing Yu, Zhongjie Zhu, Zhuoran Yang, Wei Shao

**Affiliations:** 1 Jiangsu Co-Innovation Center of Efficient Processing and Utilization of Forest Resources, Nanjing Forestry University, Nanjing 210037, China; wenyanyis@163.com; 2 College of Chemical Engineering, Nanjing Forestry University, Nanjing 210037, China; yubing1007@163.com (B.Y.); 13382367651@163.com (Z.Z.); y1693383415@163.com (Z.Y.)

**Keywords:** alginate, gelatin, porosity, antibacterial

## Abstract

In the present study, sponges with the antibiotic tetracycline hydrochloride (TCH) loaded into alginate incorporated with gelatin (G/SA) were fabricated. The G/SA sponges were characterized by scanning electron microscopy (SEM), Fourier transform infrared spectroscopy (FTIR) and thermogravimetric (TG) analysis. G/SA sponges show a three-dimensional network structure with high porosity. An excellent swelling behavior and a controlled TCH release performance are observed from G/SA sponges. Moreover, they exhibit good antibacterial activity against both Gram-positive and Gram-negative bacteria.

## 1. Introduction

As the largest organ, the skin acts as a barrier against the invasion of the human body by pathogens [1]. Once it is injured, however, it becomes particularly susceptible to bacterial infections. It was reported that mortality in burn patients associated with bacterial infections is up to 75% [2]. Therefore, wound dressing materials with infection prevention, elimination of excess exudates, air permeation and hemostasis are in high demand for the treatment of damaged skin [3,4,5].

Natural biopolymers including chitosan, alginate, cellulose, collagen, hyaluronic acid and gelatin in the form of films, sponges and fibers have been applied in wound dressing applications [6,7,8]. Among these biopolymers, sodium alginate (SA) extracted from brown seaweeds is one of the most commonly used biomaterials owing to its nontoxicity, facile production, biodegradability, low cost and biocompatibility. Therefore, SA has many applications such as biomedical, food packaging, pharmaceutical and cosmetic fields [9,10]. However, SA has some limitations in the process of non-spherical forms due to its rigid and fragile nature. Blending SA with another biopolymer is considered to be an ideal solution to overcome this drawback. SA blended with chitosan loaded with gentamicin was fabricated via the electrospinning method to be used as a wound dressing nanofibrous material with good antibacterial property and enhanced skin regeneration [6]. Chitin/chitosan and fucoidan were added to SA to develop a functional wound dressing for the repair of healing-impaired wounds [11]. A SA/aloe vera/cellulose nanocrystals composite film was prepared by a solvent casting technique and it was demonstrated to effectively inhibit the growth of *Staphylococcus aureus* bacterial colonies, thus showing potential for wound dressing applications [12].

Gelatin is a water-soluble denatured protein derived from skin, tendons or bones [13]. It possesses many advantages such as abundance, low cost, renewability, excellent film-forming ability, good absorption capacity and great biocompatibility [14]. Therefore, it finds wide application in various fields such as the food industry, water treatment, pharmaceuticals, drug delivery, scaffolds and wound dressings [15]. Gelatin-based composites exhibit great potential in wound dressings and tissue engineering since they can provide an essential microenvironment for tissue growth and excellent water absorbance capacity [16]. Gelatin/dextran–maleic anhydride composite fibers were prepared via electrospinning and photocrosslinking methods and the resulting composite fibers could support cell proliferation and adhesion, showing potential in tissue engineering [17]. A bilayered human amniotic epithelial cells and Wharton’s jelly-derived mesenchymal stem cells-laden alginate/gelatin composite hydrogel was fabricated by a 3D bioprinting method. This represents a potential preparation method for skin substitutes [18]. Konjac/gelatin hydrogel was fabricated by alkali processing and thermal treatment and it showed excellent matrine encapsulation efficiency. Moreover, matrine-loaded konjac/gelatin hydrogel could not only maintain a physiological environment beneficial to wound healing, but also inhibit the growth of bacteria on the wound surface [19]. A novel multifunctional poly(γ-glutamic acid)/gelatin hydrogel was fabricated and confirmed to be a promising wound dressing material for the wound healing [20]. Dialdehyde carboxymethylcellulose crosslinked gelatin/polyethylene glycol composite hydrogel fiber was prepared and shown to be well-suited for wound dressing applications with low cytotoxicity [21].

In this work, gelatin was incorporated into SA to enhance the shape forming properties and tetracycline hydrochloride (TCH) was then loaded to fabricate antibacterial gelatin/SA (G/SA) composite sponges. TCH is one of the most widely applied cationic antibiotics and it is effective for various bacterial infection treatments, such as periodontal, skin and bone, urinary and acnes [22,23]. The morphologies of the prepared G/SA sponges were investigated by scanning electron microscopy (SEM) and they were further characterized by Fourier transform infrared (FTIR), Thermogravimetric (TG) analysis, porosity and swelling behavior determinations. The TCH release profile was revealed by an in vitro release study in phosphate buffer saline (PBS) buffer solution at pH 7.4. Antimicrobial activity against Gram-negative *E*. *coli* ATCC 25,922 and *B. subtilis* ATCC 9372, Gram-positive *S*. *aureus* ATCC 6538 and *P. aeruginosa* ATCC 27,853 was determined by measuring the zone of inhibition.

## 2. Materials and Methods

### 2.1. Materials

Gelatin from porcine skin (type A, gel strength ~300 g Bloom) was purchased from Sigma-Aldrich (St. Louis, MO, USA). Tetracycline hydrochloride (TCH, >98%) was purchased from EKEAR Bio@Tech Co. Ltd. (Shanghai, China). SA was purchased from Aladdin Chemical Co. Ltd. (Shanghai, China). The other chemicals used in the tests were purchased from Sinopharm Chemical Reagent Co., Ltd. (Shanghai, China). All reagents were analytical grade and used as received without further purification.

### 2.2. Synthesis of G/SA Composite Sponges

Firstly, a 2% SA solution, 1% gelatin solution and 2% CaCl_2_ solution were prepared, respectively. SA solution and gelatin solution were mixed with a weight ratio of 1:1 to prepare G/SA mixture. Then 10 g G/SA mixture was poured onto a glass plate with the diameter of 60 mm, and cross-linked by CaCl_2_ solution for 24 h. TCH was dissolved in de-ionized (DI) water to achieve different concentrations of 0.005, 0.01, 0.05, 0.1 and 0.2 mg/mL, respectively. The obtained G/SA hydrogels were placed to 40 mL TCH solutions for 24 h in the dark. Then the TCH-loaded G/SA hydrogels were taken out and rinsed with DI water to remove any unloaded TCH and the excessive CaCl_2_, and freeze-dried at −40 °C for 24 h. The obtained TCH loaded sponges obtained from immersing them in different concentrations of TCH solutions are marked as G/SA1, G/SA2, G/SA3, G/SA4 and G/SA5.

### 2.3. Characterization

A JSM-7600F SEM (JEOL, Tokyo, Japan) was used to investigate the surface morphologies of G/SA and G/SA5 sponges. FTIR spectra were recorded on a Spectrum Two Spectrometer (Perkin Elmer, Akron, OH, USA). TG analysis was carried out using a model Q5000 TGA (TA Instruments, New Castle, DE, USA) with a heating rate of 10 °C/min under nitrogen atmosphere.

### 2.4. Swelling Assays and Porosity Determination

The swelling behaviors of the sponges were determined through a gravimetric method. First, the sponges were cut into round pieces with the diameter of 10 mm and their dry weights (W_0_) were accurately measured. Each sample was immersed in DI water at room temperature. The swollen sponges were withdrawn from the water and their wet weights of the swollen sponges (W_1_) was measured after the removal of excess surface water by gently blotting with a filter paper. The swelling ratio of the sponges was calculated as follows:

(1) $$\mathrm{Swelling}\;\mathrm{ratio}=\frac{W_{1}-W_{0}}{W_{0}}\times 100\%$$

The porosity was determined using an immersing method in the ethanol until saturation. The samples with the diameter of 10 mm were weighed before and after immersion into the ethanol. All testing was proceeded in triplicate. The porosity was calculated according to Equation (2):

(2) $$\mathrm{Porosity}=\frac{W_{2}-W_{1}}{\rho V}\times 100\%$$

where W_1_ and W_2_ are the weights of tested sponges before and after immersion in the ethanol, ρ is the density of ethanol and *V* is the volume of the sponge.

### 2.5. TCH Loading Calculation

TCH loadings were calculated according to the original concentration of TCH (C_0_) and the concentration of unloaded TCH (C_1_) determined by a Spectrum Two Spectrometer (Perkin Elmer, Akron, OH, USA) at the monitoring wavelength of 356 nm. TCH loadings (W) in the G/SA sponges were calculated using the following equation:

(3) $$W=\frac{C_{0}-C_{1}}{A}\times V$$

where V is the volume of total TCH solution, A is the area of the G/SA sponge.

### 2.6. TCH Release Behaviors

The TCH release behaviors were studied in 0.1 M phosphate buffer saline (PBS) buffers with pH 7.4. The sponges were cut into round pieces with the diameter of 10 mm. The tested samples were fully immersed in a beaker containing 50 mL PBS at 37 °C and placed in a dark place. At specific time points, an aliquot of 3.5 mL was collected from each solution and the absorbance was then measured at 356 nm on a Spectrum Two Spectrometer (Perkin Elmer, Akron, OH, USA). An equivalent volume of fresh PBS buffer was replaced into the system after each sampling to maintain constant medium volume. Thus, the concentrations of released TCH obtained at different times can be calculated. As a result, the cumulative released TCH amounts can be calculated accordingly. The experiments were performed in triplicate.

### 2.7. Antibacterial Activity

The antibacterial activities of G/SA sponges were investigated against Gram-negative *E*. *coli* ATCC 25,922 and *B. subtilis* ATCC 9372, Gram-positive *S*. *aureus* ATCC 6538 and *P. aeruginosa* ATCC 27,853 by disk diffusion method. G/SA sponges were cut into round shapes with 10 mm diameter and sterilized by ultraviolet lamp for 60 min. Lawns of test bacteria (about 1 × 10^5^ CFU/plate) were prepared on TSA. The sterilized samples were then carefully placed upon the lawns. The plates were placed in a 37 °C incubator for 24 h. Then inhibitory action was determined by measuring the diameter of the inhibition zones.

### 2.8. Statistical Analysis

The obtained data are expressed as mean ± standard error (SE). Statistical differences were evaluated using a Student’s t-test. A *p*-value of < 0.05 was considered to be statistically significant.

## 3. Results and Discussion

### 3.1. Surface Morphology

**Figure 1:**
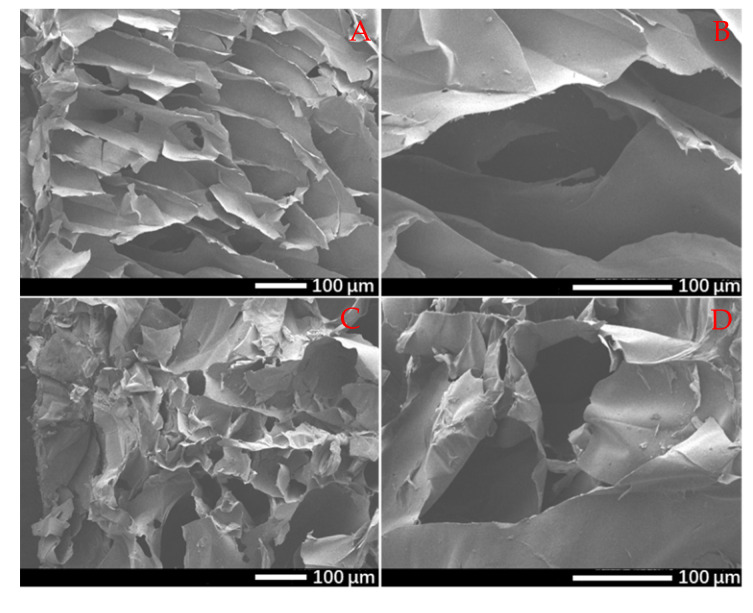
SEM images of G/SA (**A**,**B**) and G/SA5 (**C**,**D**) sponges.

The exudate absorption capacity is directly affected by the surface morphology and porosity of polymer-based wound dressings, so the morphologies of G/SA and TCH-loaded G/SA (G/SA5) sponges were studied using SEM (Figure 1). Figure 1A,B show the cross-sectional morphologies of G/SA sponge with different magnifications. It can be clearly seen that G/SA sponge exhibits a highly porous three-dimensional network structure with interconnected pores throughout the hybrid sponge. After TCH was loaded into the sponge, small difference in pores shape and size can be found although G/SA5 sponge still keeps its 3D structure, as shown in Figure 1C,D.

**Figure 2:**
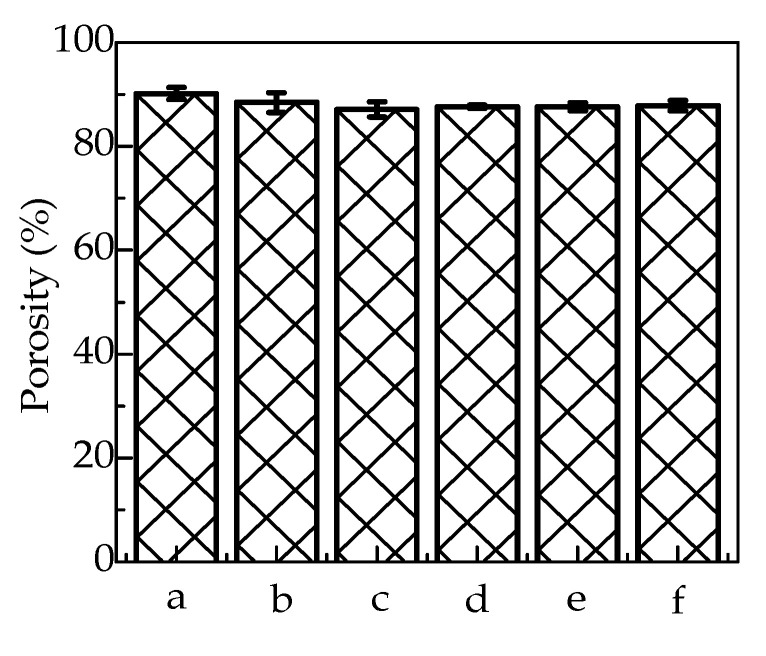
Porosities of G/SA and TCH loaded G/SA sponges (a:G/SA, b:G/SA1, c:G/SA2, d:G/SA3, e:G/SA4 and f:G/SA5).

Figure 2 shows the porosities of G/SA and TCH loaded G/SA sponges. The G/SA sponge shows a relatively high porosity of 90.2%. After TCH was loaded into G/SA sponges, a slight decrease in porosity to a range of 87.1–88.5% can be observed. TCH loadings of G/SA composite sponges are listed in Table 1. It can be clearly found that TCH loading increases with the increase of initial TCH concentration. These high porosities benefit for exudates absorbing, making the fabricated sponges show great potentials in wound dressings application.

### 3.2. Thermal Properties

**Figure 3:**
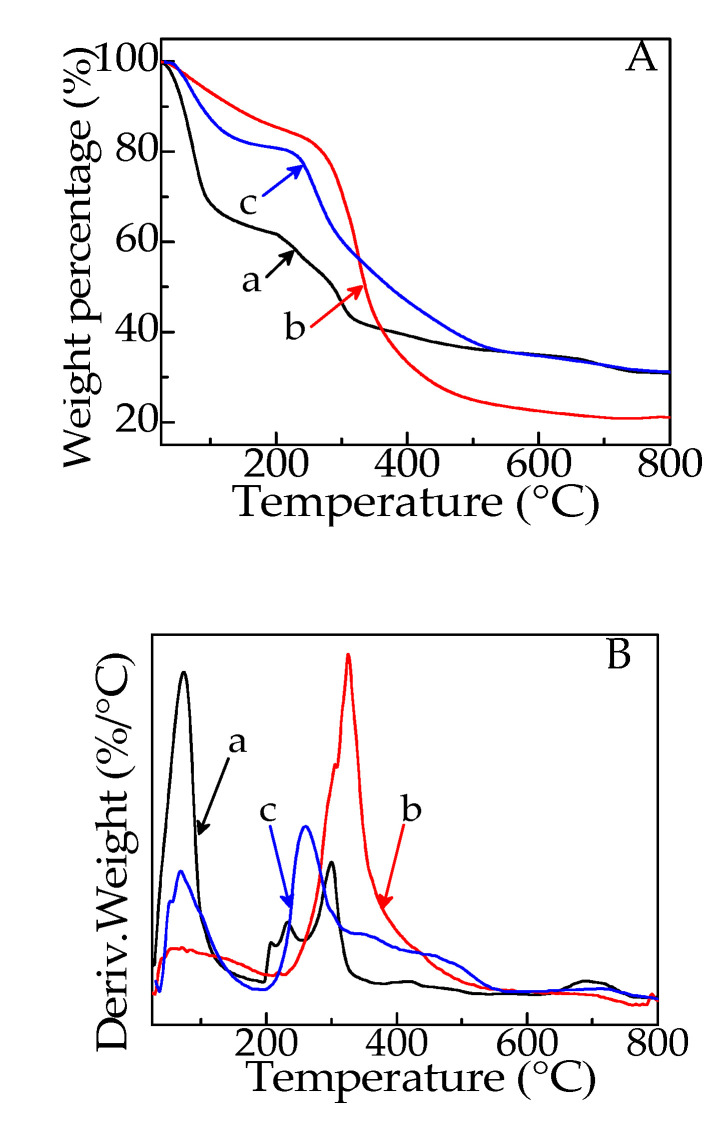
TG (**A**) and DTG (**B**) curves of SA (a), gelatin (b) and G/SA5 (c).

The TG and derivative thermogravimetric (DTG) curves of SA, gelatin and G/SA5 are shown in Figure 3. Three weight loss stages were found in the TG curve of SA (curve a in Figure 3A). The first weight loss stage occurs below 120 °C, which is attributed to the evaporation of absorbed water. The second weight loss occurred in the range of 200–350 °C involving the degradation of main backbone of SA and the formation of sodium carbonate. The third weight loss stage starting from 350 °C to 730 °C is the decomposition of both SA and sodium carbonate [24,25]. The residue of SA is 30.8%, corresponding to the formed carbon dioxide and calcium oxide [26]. Thermal degradation of gelatin occurs in two stages (curve b in Figure 3A). The first stage occurs from room temperature up to 180 °C and the second stage occurs between 180 °C and 370 °C corresponding to the degradation of protein [27].

In the case of G/SA5 sponge (curve c in Figure 3A), two weight loss stages were shown. The first one is the loss of adsorbed water which occurs below 200 °C. The second one is the complex decomposition of SA, gelatin, sodium carbonate and TCH in the range of 200–780 °C. The residue of G/SA5 is 31.2%, which is higher than those of SA and gelatin (21.1%) due to the existence of TCH. Therefore, TCH are demonstrated successfully to be incorporated into G/SA sponges.

### 3.3. FTIR Analysis

**Figure 4:**
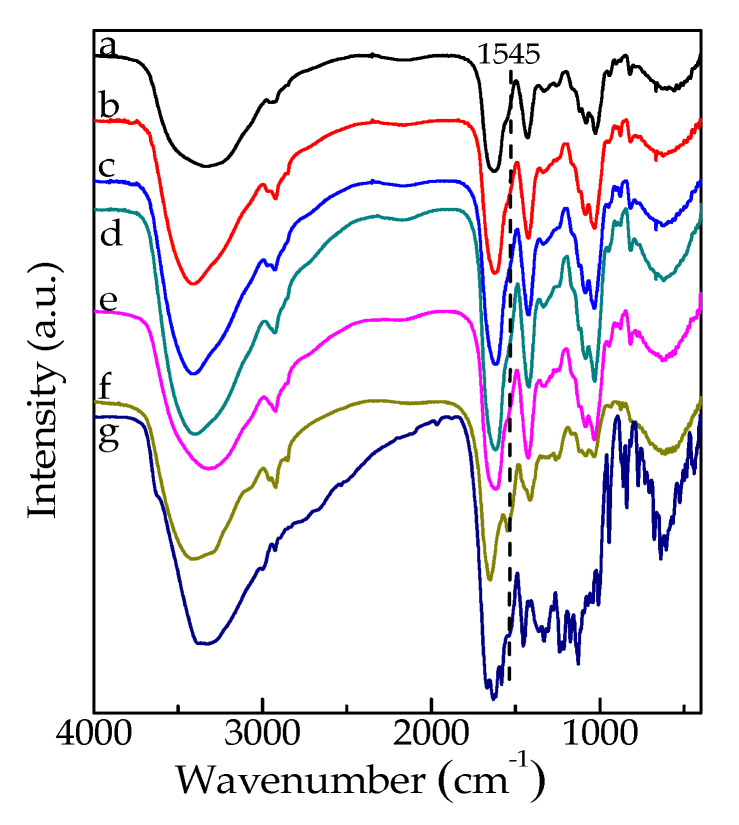
FTIR spectra of G/SA (a), G/SA1 (b), G/SA2 (c), G/SA3 (d), G/SA4 (e), G/SA5 (f) sponges and TCH (g).

FTIR spectra of G/SA, G/SA1, G/SA2, G/SA3, G/SA4, G/SA5 and TCH are given in Figure 4. In the case of G/SA (curve a), characteristic bands located at 3328 cm^−1^, 1559 cm^−1^ correspond to the stretching of -NH and -OH groups, and the N-H deformation (amide II) of gelatin [28]. Moreover, the peak at 1624 cm^−1^ is assigned to the C=O stretching (amide I) of gelatin and the asymmetric COO stretching vibration of SA. The absorption band at 1419 cm^−1^ corresponds to the symmetric COO stretching vibration of SA [13,29]. The asymmetric and symmetric COO stretching vibrations of the reported SA without any cross-linking are located at 1612 and 1417 cm^−1^, respectively [30]. They show a blue shift due to the formation of ionic bonding between -COO^-^ and Ca^2+^ in this study. In the FTIR spectrum of TCH (curve g), the peaks located at 1665 cm^−1^, 1617 cm^−1^, 1590 cm^−1^, 1545 cm^−1^ and 1440 cm^−1^ are assigned to the C=O vibration of amide I, the C=O vibration of A-ring, the C=O vibration of C-ring, the N-H deformation of amide II and the C=C vibration of aromatic ring, respectively [31]. For TCH-loaded G/SA sponges (curves b–f), the intensity of peak at 1545 cm^−1^ increases with TCH loading raising although most characteristic peaks of TCH overlaps with the peaks of G/SA. Thus, the existence of TCH in the G/SA sponges is verified.

### 3.4. Swelling Behaviors In Vitro

**Figure 5:**
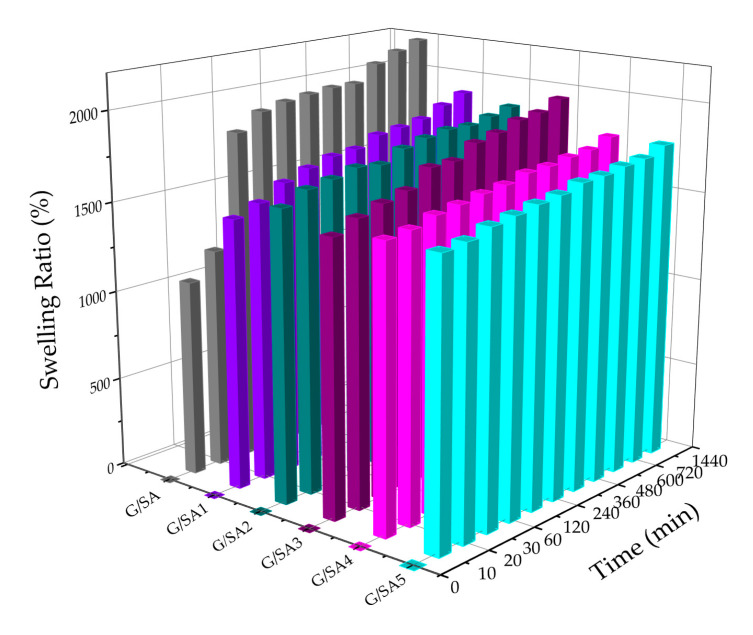
Swelling behaviors of G/SA composite sponges.

The water absorbing and holding abilities are essential characteristics for polymer-based wound dressings which facilitate the adsorption of exudates, body fluids or metabolites. Figure 5 shows the dynamic swelling performance of G/SA composite sponges. The swelling ratio of G/SA sponge is 2190% after 24 h, which is the highest swelling ratio among the tested sponges. SA has many hydrophilic groups including carboxylic and hydroxyl groups, and gelatin also contains carboxylic and hydroxyl groups as well as amino groups, leading to a high swelling performance. Meanwhile, TCH loaded G/SA sponges show slightly reduced swelling performance with the swelling ratios ranging from 1780 to 1960% after 24 h. This phenomenon could be due to the fact that the porosities of sponges were slightly decreased after being loaded with TCH.

### 3.5. TCH Release Behavior

**Figure 6:**
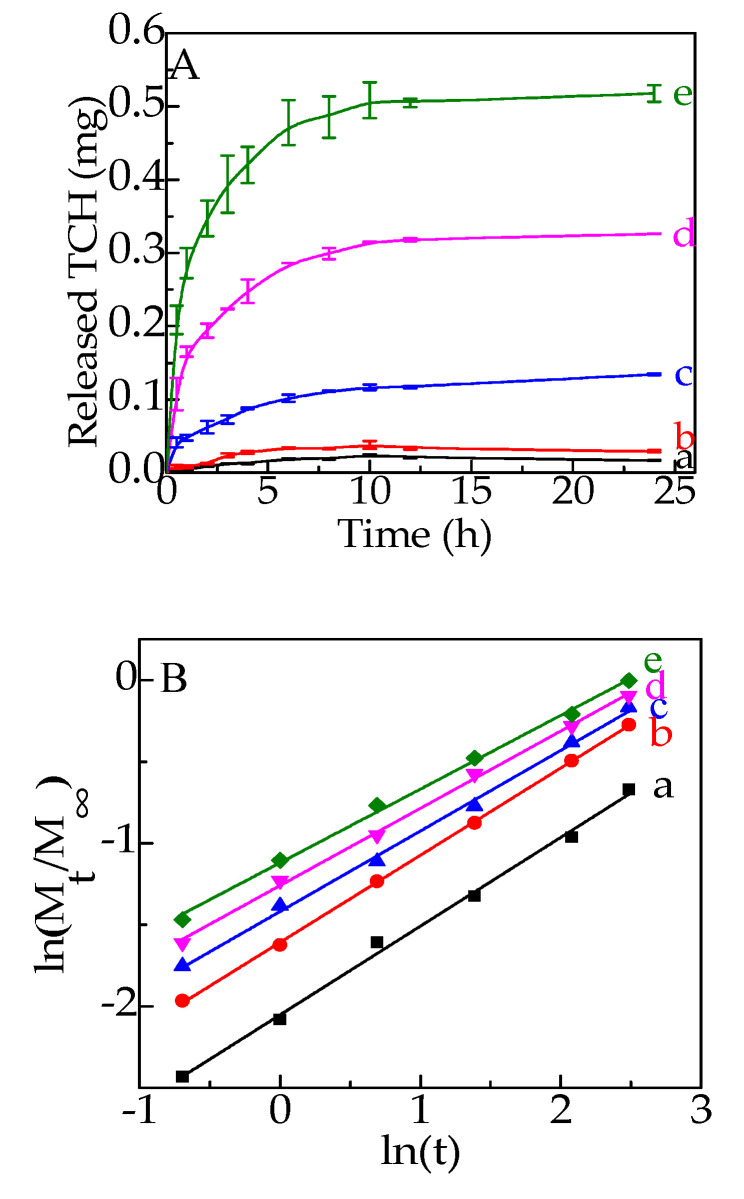
TCH release behavior of G/SA sponges (**A**) and plots of ln(Mt/M) versus ln(t) (**B**). (a:G/SA1, b:G/SA2, c:G/SA3, d:G/SA4, e:G/SA5).

The TCH release behaviors were determined in 0.1 M PBS buffer at pH 7.4 and the result is shown in Figure 6A. A burst release behavior was displayed in the first 1 h for the TCH-loaded G/SA sponges and this behavior could be probably due to the accumulation of TCH on the surface of G/SA sponges. Then a gradual increased release behavior for the G/SA composite sponges was shown and the release behavior lasts for 12 h. Moreover, the released amount is positively related to the TCH loadings in the G/SA sponges.

Thus, G/SA composite sponge is considered to be a good TCH carrier with controlled release performance, demonstrating its potential wound dressing application in the treatment of infections and inflammation.

Four mathematical models, including zero order, first order, Higuchi and Korsmeyer-Peppas models were used to fit the TCH release profiles. The obtained results are shown in Table 2. The best fitting mathematical model for the TCH release mechanism was determined by the calculated correlation coefficient (R^2^). It can be found that the Korsmeyer-Peppas model is the best fitting one, which is a semi-experimental exponential function equation considering diffusion of drug and erosion of polymer matrix [16]. The plots of ln(Mt/M) versus ln(t) using Korsmeyer-Peppas model are displayed in Figure 6B. The diffusional constant n indicates the drug release mechanism. In this study, n values lie between 0.45 and 0.89, suggesting the TCH release behavior follows non-Fickian diffusion, which corresponds to the combined effects of the drug releases by dissolution, diffusion and erosion of matrix.

### 3.6. Antibacterial Activity

**Figure 7:**
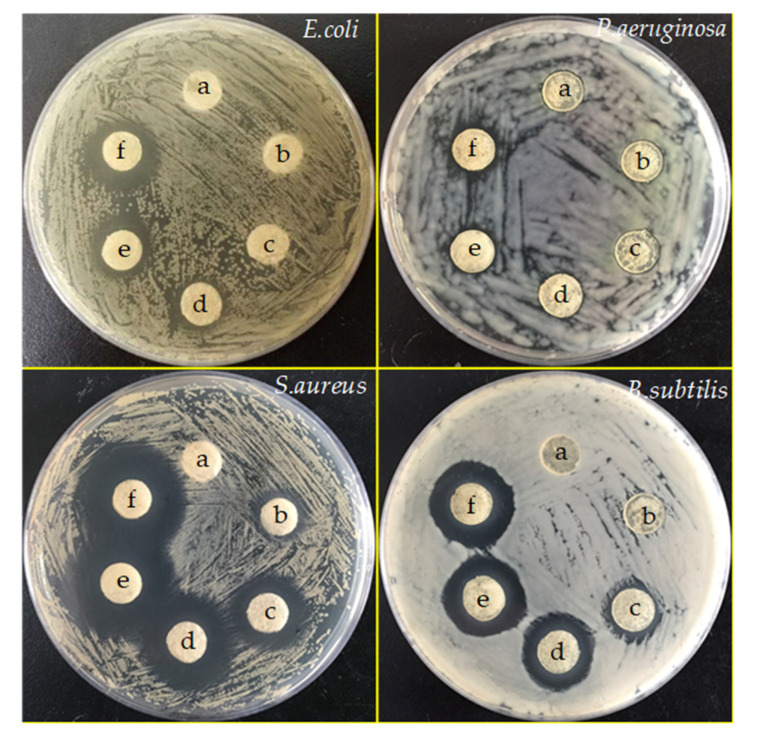
Inhibition zone pictures of G/SA composite sponges: G/SA (a), G/SA1 (b), G/SA2 (c), G/SA3 (d), G/SA4 (e), G/SA5 (f) sponges.

**Figure 8:**
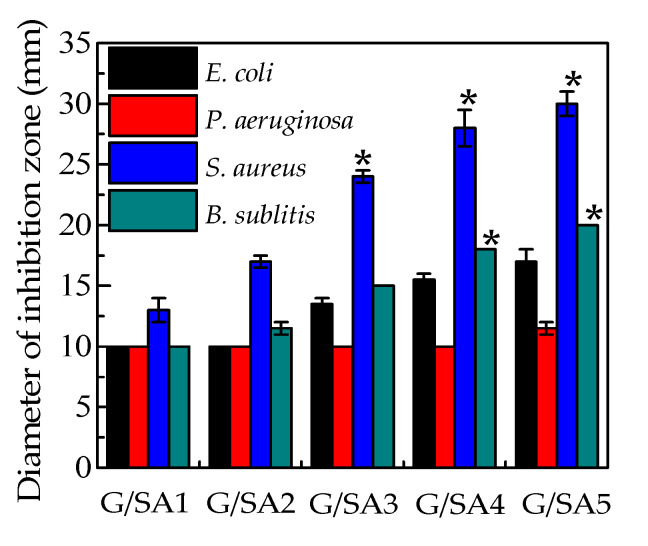
Diameters of inhibition zone of G/SA composite sponges against different strains. (* *p* < 0.05).

The antibacterial activities of G/SA composite sponges towards Gram-negative *E*. *coli* ATCC 25,922 and *B. subtilis* ATCC 9372, Gram-positive *S*. *aureus* ATCC 6538 and *P. aeruginosa* ATCC 27,853 were investigated by the disc diffusion method. The antibacterial activity is determined by measuring the clear zone of inhibition around the samples after 24 h incubation (Figure 7). For G/SA without any TCH loading, no inhibition zones were observed around the tested four strains, implying that G/SA sponge does not have any antibacterial activities. While, G/SA composite sponges have different levels of antibacterial activities. The measured average diameters of inhibition zones are shown in Figure 8. It can be clearly seen that the inhibition zone increases with the increase of TCH loading in the G/SA sponge. Moreover, G/SA composite sponges show a good antibacterial activity against *E. coli*, *S*. *aureus* and *B. subtilis*. However, little antibacterial activity against *P. aeruginosa* was shown. The differences in the antibacterial activity could be due to the susceptibility difference against different strains of the loaded TCH. Therefore, G/SA composite sponges show good antibacterial activities, which makes them have great potentials in the antibacterial wound dressing materials applications.

## 4. Conclusions

In summary, TCH-loaded G/SA sponges were successfully synthesized and they display good swelling behavior and high porosity. G/SA composite sponges exhibit three-dimensional network structures, which help the G/SA sponges to facilitate TCH loading. The TCH-loaded G/SA sponges show controlled release performance and good antibacterial effects against *E. coli* ATCC 25,922 and *B. subtilis* ATCC 9372 and *S. aureus* ATCC 6538. Thus, the fabricated G/SA sponges show potential as promising substrates for drug delivery applications in wound dressing and other biomedical applications.

## Figures and Tables

**Table 1:** TCH loadings of G/SA composite sponges.

	Initial TCH Concentration (mg/mL)	TCH Loading (mg/cm^2^)
G/SA1	0.005	0.081
G/SA2	0.01	0.100
G/SA3	0.05	0.516
G/SA4	0.1	0.873
G/SA5	0.2	1.463

**Table 2:** K, n and R^2^ values obtained from the mathematical modeling.

Type of Model	Name of Samples	Fitting Parameters		
		K	n	R^2^
Korsmeyer-Peppas M_t_/M_∞_ = Kt^n^	G/SA1	0.12836	0.54398	0.99805
	G/SA2	0.200118	0.53455	0.99986
	G/SA3	0.241837	0.49382	0.9987
	G/SA4	0.283569	0.47373	0.99919
	G/SA5	0.325983	0.452	0.99866
Zero-order M_t_/M_∞_ = Kt	G/SA1	0.06694	/	0.91416
	G/SA2	0.06119	/	0.69879
	G/SA3	0.07117	/	0.97968
	G/SA4	0.04232	/	0.94553
	G/SA5	0.04489	/	0.87162
First-order M_t_/M_∞_ = 1 − e^−Kt^	G/SA1	0.24059	/	0.98953
	G/SA2	0.31313	/	0.92621
	G/SA3	0.41652	/	0.90066
	G/SA4	0.7134	/	0.88187
	G/SA5	0.91565	/	0.8789
Higuchi M_t_/M_∞_ = Kt^1/2^	G/SA1	0.2986	/	0.9315
	G/SA2	0.36825	/	0.87818
	G/SA3	0.2243	/	0.96647
	G/SA4	0.22741	/	0.94
	G/SA5	0.20267	/	0.90434
